# The Hybridge Technique: A Combined Technique of Suture Bridge and Tension Band for an Arthroscopic Eco-Responsible Rotator Cuff Repair

**DOI:** 10.1016/j.eats.2022.08.025

**Published:** 2022-11-18

**Authors:** Vincent Martinel, Geoffroy Nourissat, Johannes Barth, Bruno Zipoli, Nicolas Bonnevialle, the Green Shoulder Circle

**Affiliations:** a Groupe Orthopédie Ormeau Pyrénées, ELSAN - Polyclinique de l’Ormeau, Tarbes, France; b Clinique Maussins-Nollet, Sorbonne Université, Paris, France; c Département de chirurgie orthopédique, Centre ostéoarticulaire des cèdres, Parc Sud Galaxie, Echirolles, Grenoble, France; d Service orthopédie et traumatologie, centre hospitalier de Dax, Dax, France; e Département d’orthopédie traumatologie CHU de Toulouse, place du docteur Baylac, Hôpital Riquet, Toulouse, France; h Green Shoulder Circle, Lourdes, France, France

## Abstract

Arthroscopic rotator cuff repair is mainly based on 2 proven biomechanical concepts: suture bridge and tension band. This Technical Note describes the use of a combination of these 2 techniques to repair extensive lesions with only 3 anchors. Besides being less expensive, the use of a limited number of anchors is part of a global medicoeconomic and eco-responsible approach to our surgical activities.

Numerous techniques have been described for rotator cuff repair. Various biomechanical studies have confirmed the reliability of both double-row suture-bridge fixation^1^ and single-row tension band (TB).^2^ Although the latter requires fewer anchors, it may result in decreased contact at the footprint during tendon repair.^3^ In contrast, the suture-bridge technique, associated with biceps tenodesis, may require 5 or more anchors with no clear clinical benefit or improved healing.

Several recent studies have discussed the impact of health care on greenhouse gas emissions (8.5% of total emissions in the United States, 6% in France), with one-half of these associated with operating room activities.^4^^,^^5^ To participate in the reduction of the carbon footprint, it would be reasonable to develop techniques that reduce the number of anchors used in each patient with a similar clinical and anatomical result. This Technical Note describes a hybrid fixation technique that combines double-row suture-bridge and TB for repair of a large superior cuff tear (Type C or D of Colin’s classification)^6^ with 2 first-row anchors and a single knotless second-row anchor. It may also be combined with a biceps tenodesis.

## Surgical Technique (With Video Illustration)

### Preoperative Evaluation

The hybridge technique is indicated in patients with a symptomatic posterosuperior rotator cuff lesion, without risk factors for poor healing: nonsmokers, no hypercholesterolemia. Magnetic resonance imaging or computed tomography arthroscan is necessary to evaluate the size of the lesion in the anteroposterior plane, which should ideally be between 1.5 and 3.5 cm for this technique. For smaller lesions, a single first-row anchor or a TB with a single lateral anchor should be chosen. For larger lesions, an additional number of anchors is recommended. In the frontal plane, the tendon retraction and the critical shoulder angle measurement also are assessed on imaging to determine whether it will be possible to use a spinal needle as a suture pass intraoperatively. Finally, the muscular fatty infiltration index should be measured, ideally less than 2.

### Surgery

Demonstration of arthroscopic rotator cuff repair of a left shoulder using the Hybridge technique is presented in Video 1.

The advantages and disadvantages of the technique are listed in Table 1 and pearls and pitfalls in Table 2.

Surgery is performed under interscalene block and general anesthesia. The patient is placed in the lateral decubitus position, but the scope will be turned 90° to obtain a vertical view like in the beach-chair position. The upper limb is stabilized with a hydraulic arm (AssistArm; ConMed, Largo, FL). A transparent operating field is created after the skin has been prepared. Two portals are used: a posterior portal (PP), mainly for the arthroscope, and a mainly instrumental anterolateral portal (ALP).

**Table 2:** Pearls and Pitfalls of the Hybridge Technique

Pearls	Pitfalls
• Draw the acromion on the skin to facilitate positioning of the spinal needle. • To work without a cannula, retrieve the 2 strands in one pass just before tying them. • Leave the punch tap in place in the bone when mounting the second-row anchor and the strands to keep the axis. • Turn the scope so that the punch-tap is vertical to the screen before removing, and screw in the anchor on exactly the same axis (12 o'clock technique). • Always use a second-row anchor with a diameter that is larger than the pinch-tap used (1 or even 2 mm depending on the bone quality).	• Use of the spinal needle impossible with a retracted cuff or with high CSA. The use of an automatic plier is recommended. • Cutting the knotted strand of the first-row anchors instead of the traction strand and risk of knot failure.

CSA, critical shoulder angle.

**Table 1:** Advantages and Disadvantages of the Hybridge Technique

Advantages	Disadvantages
• Only 1 knotless second row-anchor instead of the usual 2 • Less expensive and less wasteful • Reusable screwdriver • Simplified instrumentation • Precise positioning of the strands on the articular surface of the tendon for optimal contact with the anatomical footprint.	• Requires knot tying • Learning curve for the transcutaneous spinal needle points

First, the subacromial space is explored with a scope in the PP, with a bursectomy to obtain a view of the entire superficial aspect of the cuff. Once the lesion is identified, the lateral part of the footprint and tendon tear are debrided.

**Fig 1:**
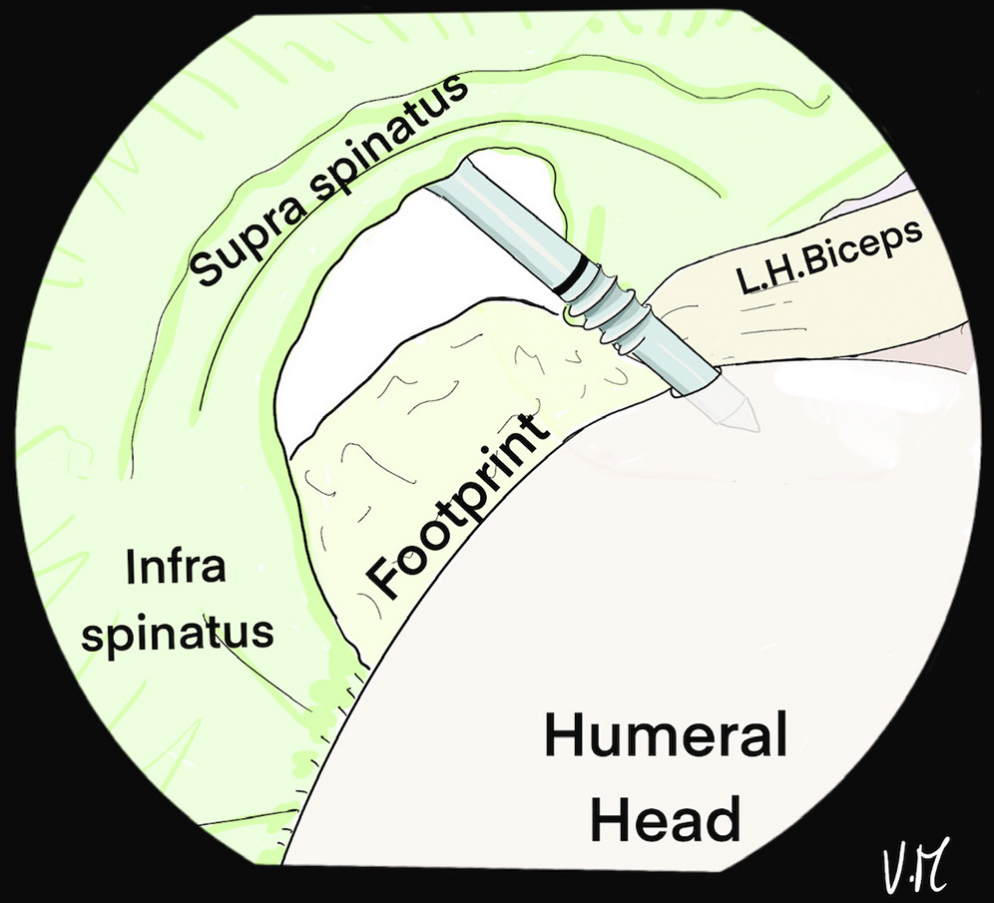
Drawing of an arthroscopic glenohumeral view from the posterior portal on a left shoulder in the beach-chair position. The deep layer of the rotator cuff is visualized (green). A 4.5-mm punch tap is inserted through the lesion at the anterior part of the footprint, behind the long head of the biceps.

**Fig 2:**
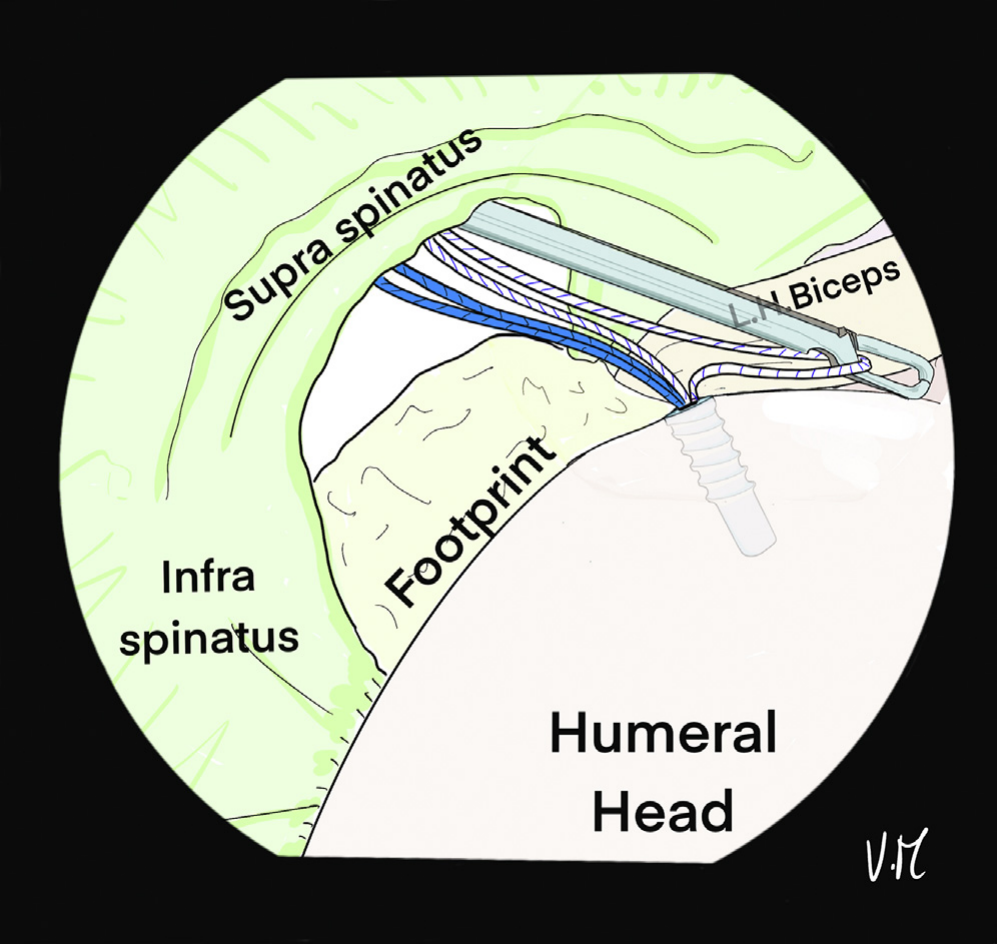
Drawing of an arthroscopic glenohumeral view from the posterior portal on a left shoulder in the beach-chair position. A 4.5-mm FIXIT anchor (SBM; Lourdes, France) has been inserted in the anteromedial position. The white suture is placed under the long head of the biceps to perform intraarticular biceps tenodesis.

**Fig 3:**
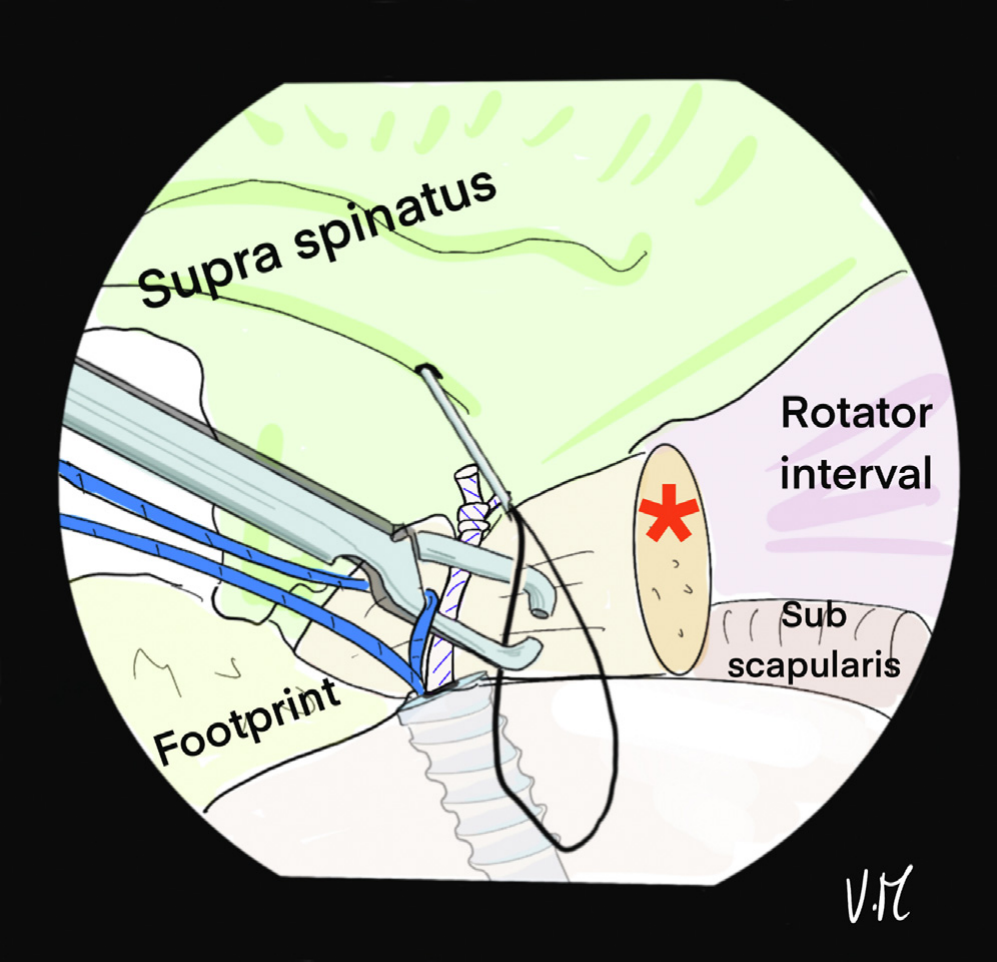
Drawing of an arthroscopic glenohumeral view from the posterior portal on a left shoulder in the beach-chair chair position. Intra-articular biceps tenodesis (∗) has been performed with a single knot. A percutaneous 22-gauge spinal needle with double monofilament is inserted to create a loop at the anterior part of the supraspinatus lesion. Intra-articular control makes it possible to identify the rotator cable at the distal end of the supraspinatus and choose exactly where to pass the suture.

**Fig 4:**
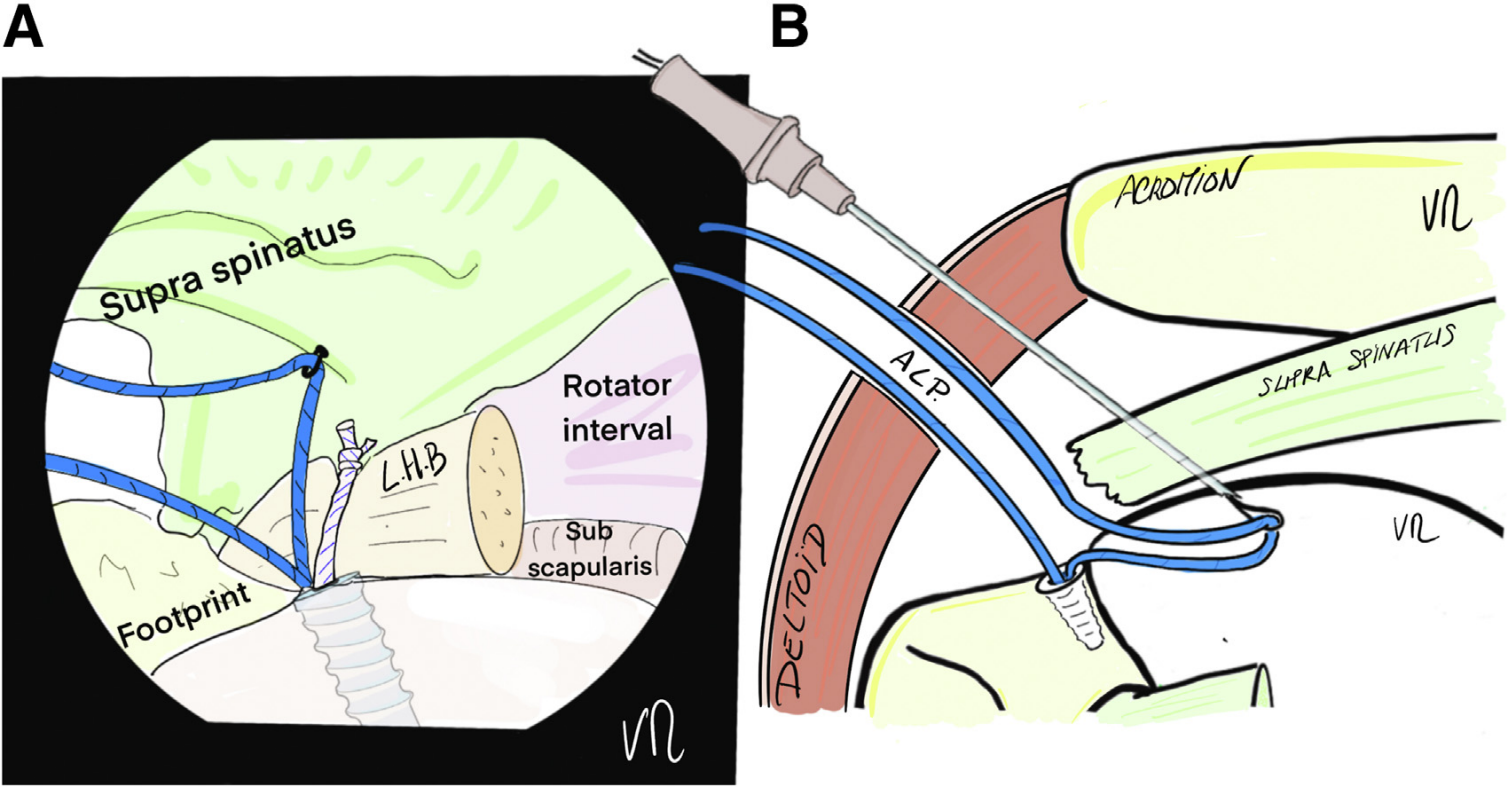
(A) Drawing of an arthroscopic glenohumeral view from the posterior portal on a left shoulder in the beach-chair position. The first anterior thread is passed from deep to the surface of the tendon by pulling on the monofilament. (B) Illustration of cross-section of a shoulder. The spinal needle with monofilament loop goes through the skin, the deltoid muscle and the supraspinatus tendon, while the suture passes through the anterolateral portal. (LHB, long head of biceps.)

**Fig 5:**
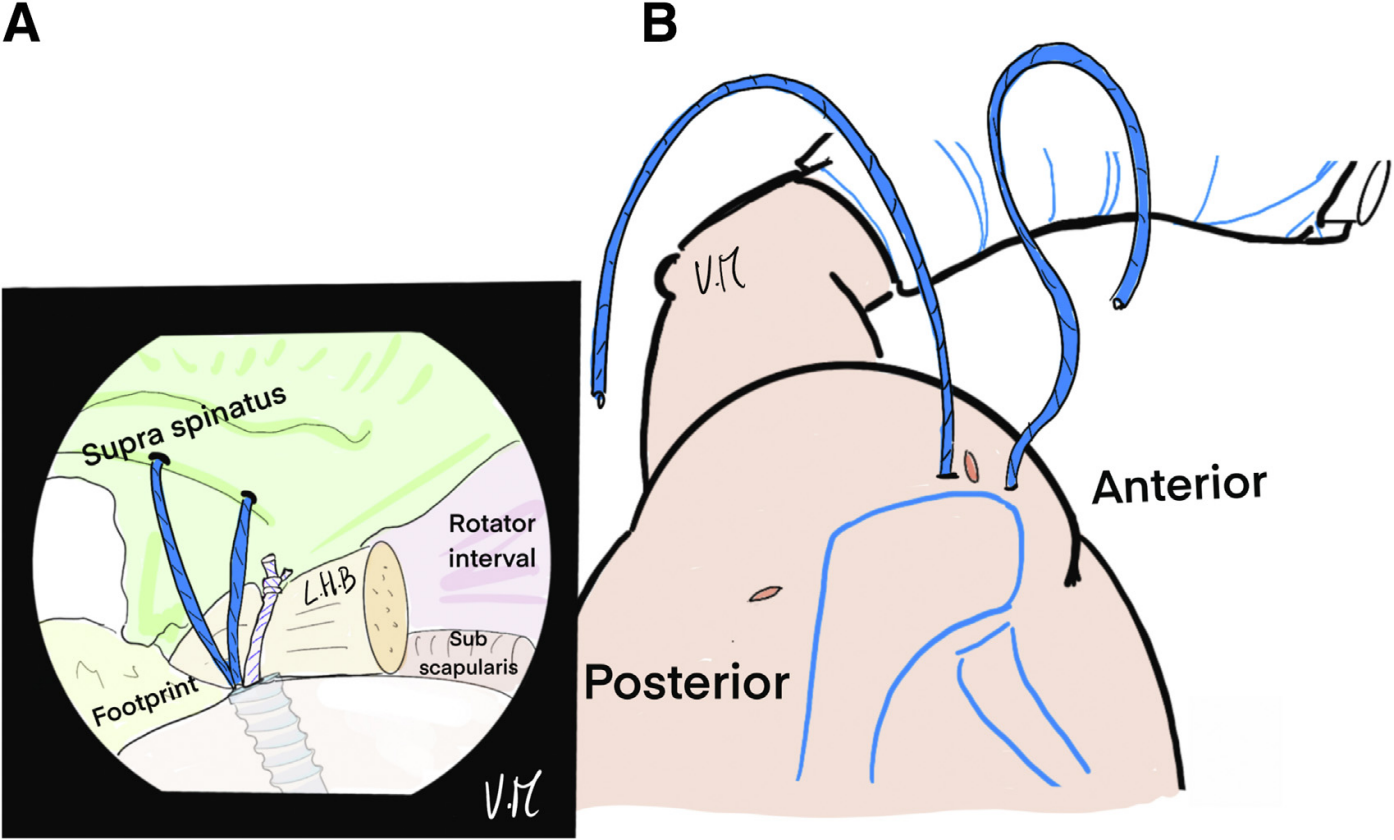
(A) Drawing of an arthroscopic glenohumeral view from the posterior portal on a left shoulder in the beach-chair position. The second strand is passed in the same way, 5 to 8 mm behind the first. (B) Drawing of a superior view of the patient’s left shoulder—the 2 strands are transcutaneous, exiting on the lateral edge of the acromion, leaving the anteromedial portal free. (LHB, long head of biceps.)

The scope is positioned in the glenohumeral joint through the PP to visualize the deep surface of the cuff. The bone is shaved at the footprint and a first 4.5-mm FIXIT anchor (SBM; Lourdes, France) is placed anteromedially in line with the biceps tendon gutter (Fig 1). Biceps tenodesis is performed with the first suture and a simple knot (Fig 2). A loop is created with a 22-gauge spinal needle and double monofilament to be used as a suture passer to successively pass the 2 strands of the second suture from the anchor to the anterior supraspinatus (Figs 3, 4, and 5A). The intra-articular view provides good visualization of the rotator cable to optimize penetration of the sutures on the articular surface of the tendon, to restore the anatomical footprint, and for optimal contact and adequate tension on the cuff during reduction. Precise spacing of 5 to 8 mm between the 2 strands is also possible. The landmark on the skin for optimal needle placement is just in contact with the lateral edge of the acromion (transcutaneous) (Fig 5B).

**Fig 6:**
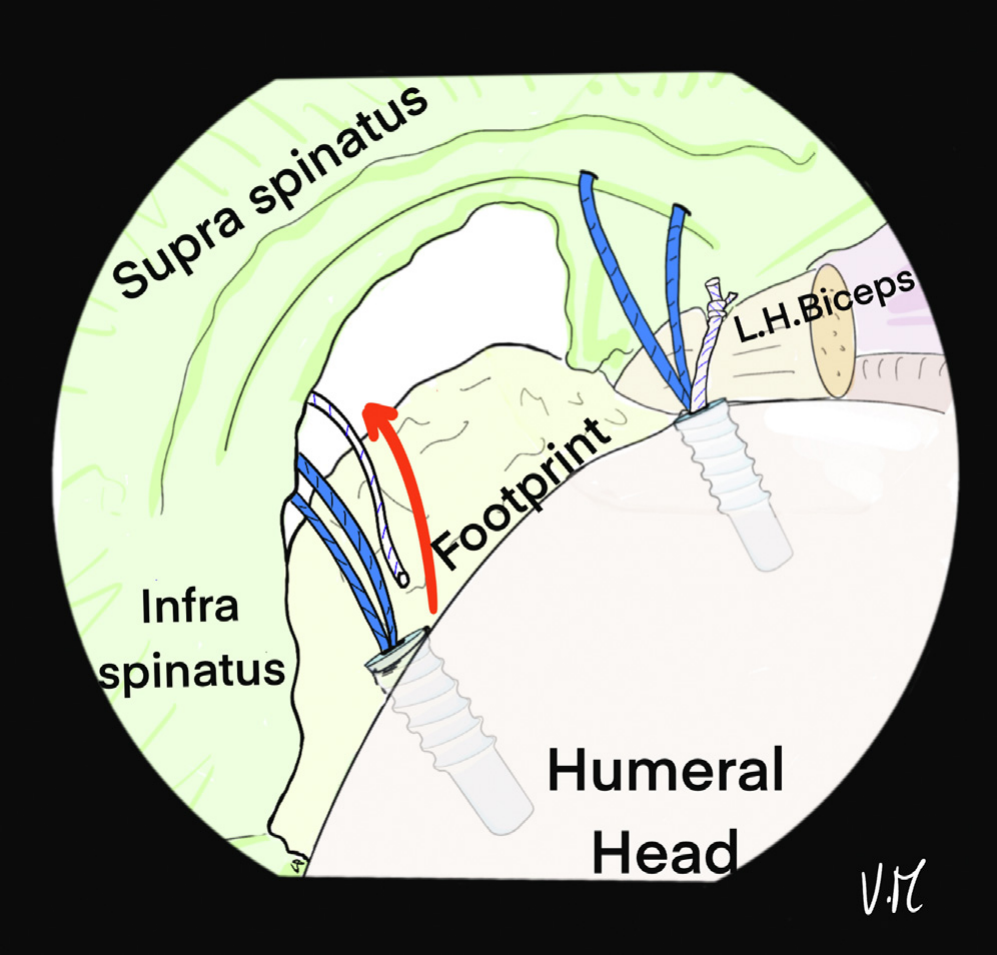
Drawing of an arthroscopic glenohumeral view from the posterior portal on a left shoulder in the beach-chair position. A second posteromedial 4.5-mm FIXIT anchor (SBM; Lourdes, France) is inserted at the footprint at the most posterior part of the lesion, then the white wire is removed and put aside. (L.H., long head.)

**Fig 7:**
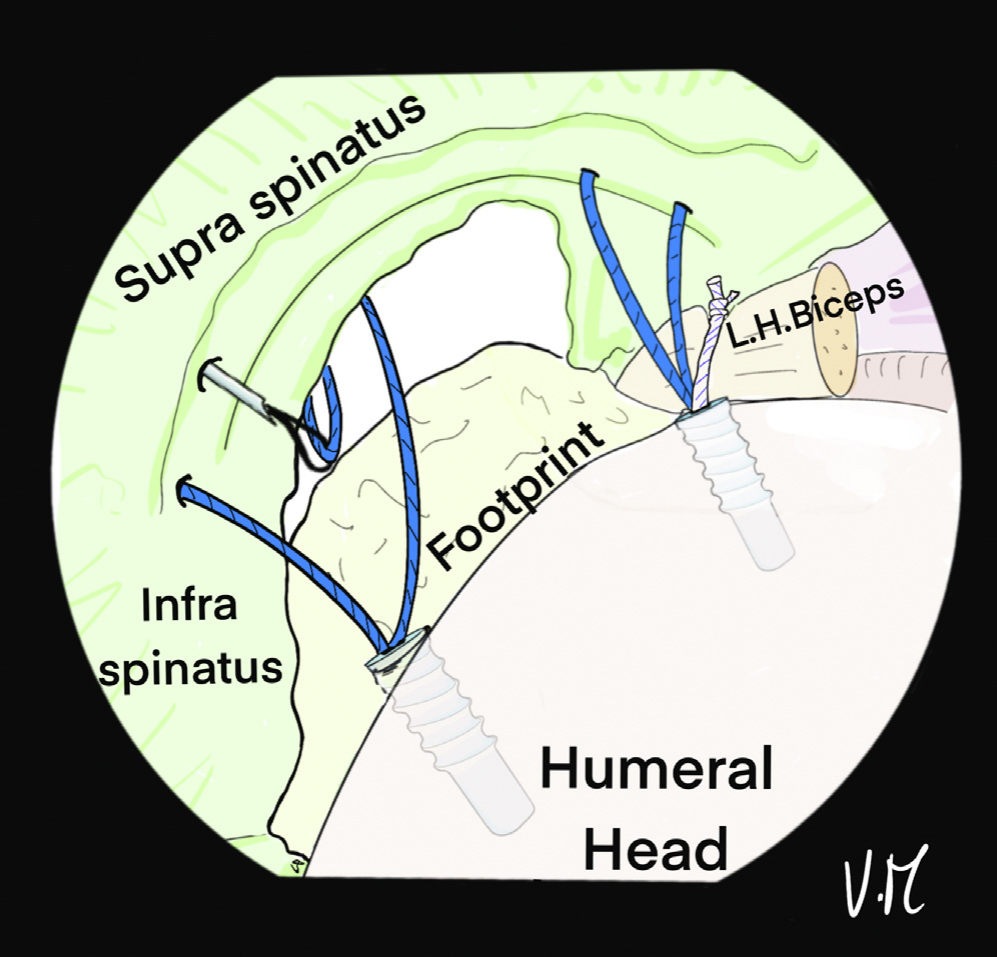
Drawing of an arthroscopic glenohumeral view from the posterior portal on a left shoulder in the beach-chair position. The first blue strand is passed at the most posterior part of the lesion and the second, 5-8 mm anterior to the first, using a 22-gauge spinal needle and a transcutaneous monofilament suture. (L.H., long head.)

A second 4.5-mm FIXIT anchor (SBM) is placed at the most posterior part of the tear at the medial footprint and 1 of the 2 sutures is removed and put aside for the TB (Fig 6). The 2 threads of the second sutures are again passed through the posterior part of the lesion and spaced 5 to 8 mm apart (Fig 7).

**Fig 8:**
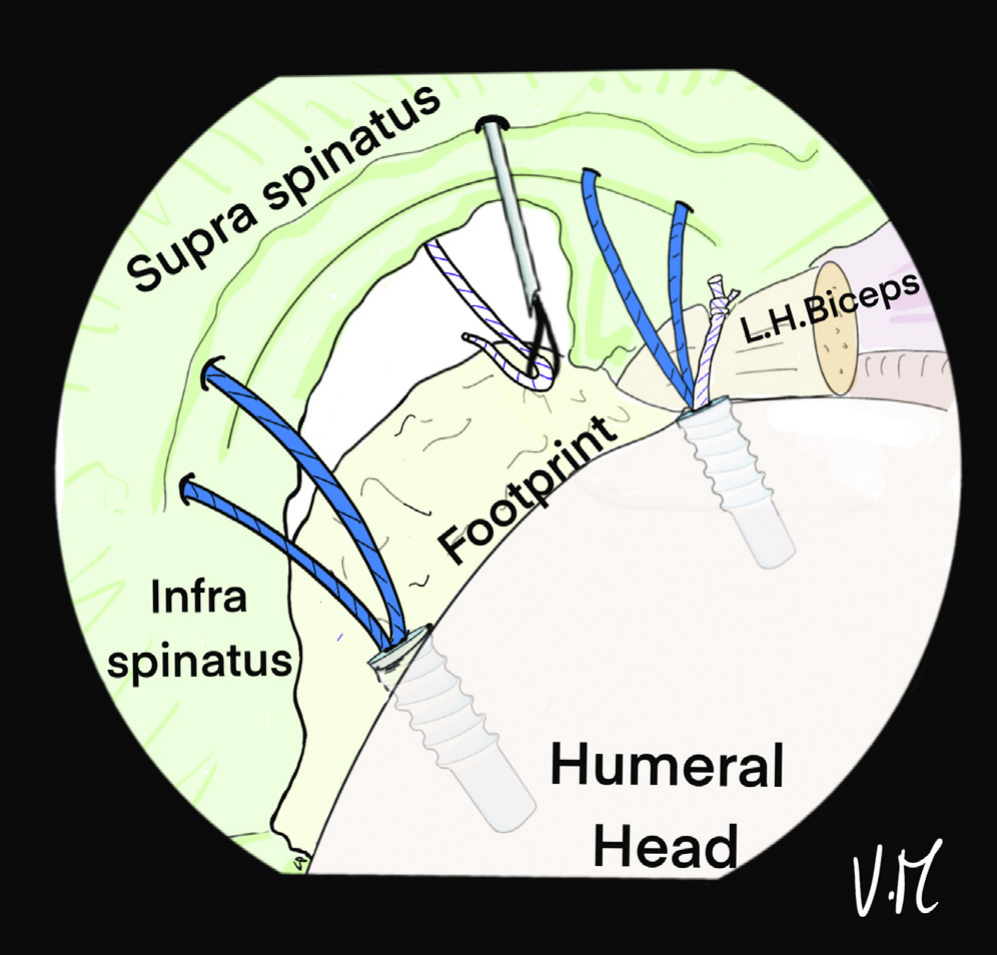
Drawing of an arthroscopic glenohumeral view from the posterior portal on a left shoulder in the beach-chair position. A 22-gauge spinal needle is passed transcutaneously to the deep surface of the supraspinatus tendon, ideally medial to the rotator cable. It is used to pass the white suture that was put aside. (L.H., long head.)

**Fig 9:**
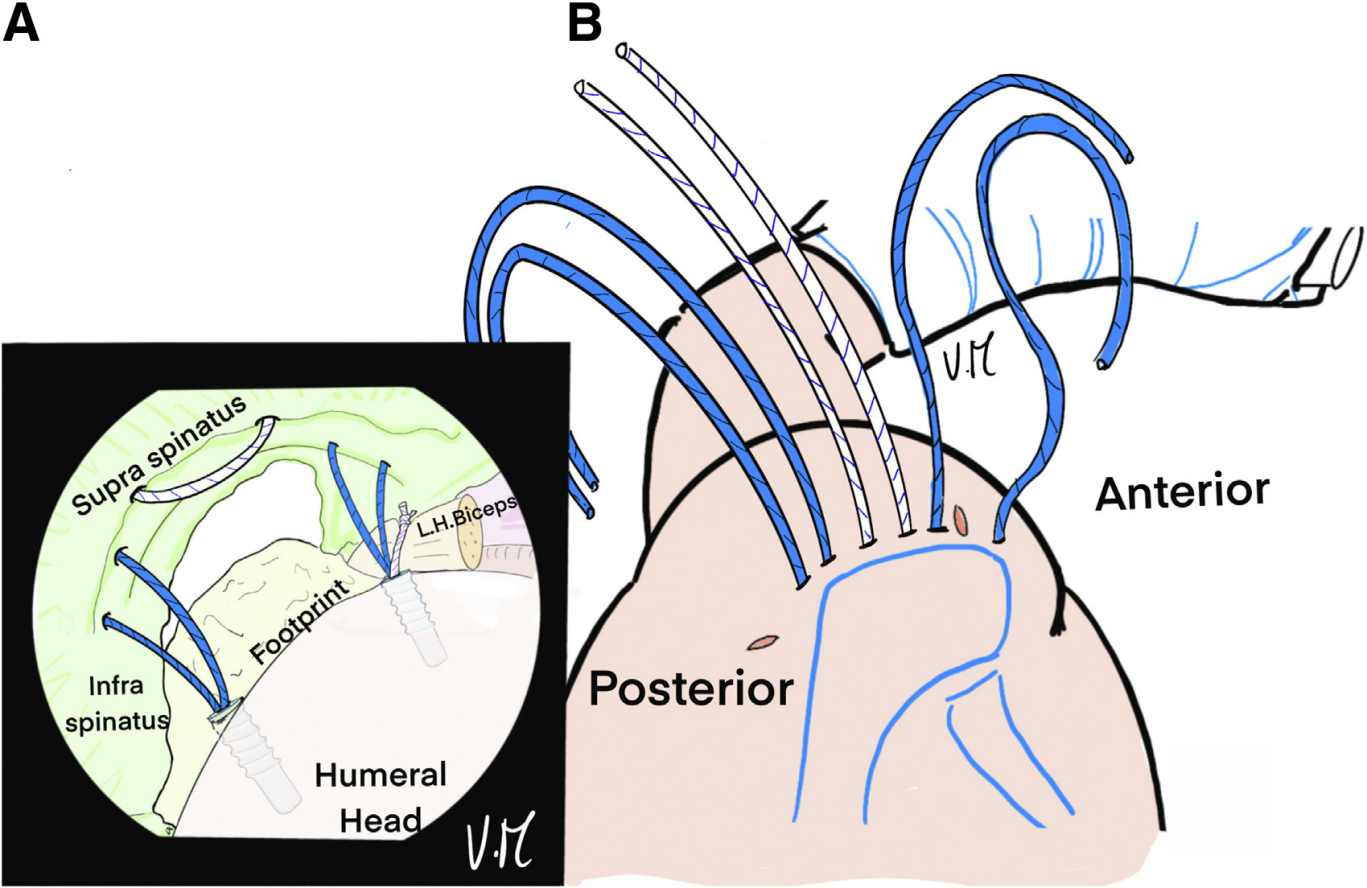
(A) Drawing of an arthroscopic glenohumeral view from the posterior portal on a left shoulder in the beach-chair position. Final appearance after the 6 strands have been passed. The white suture is U-shaped, passing in the middle of the deep part of the lesion to create the central tension band. (B) Drawing of a superior view of the patient’s left shoulder: the 6 strands are transcutaneous, in contact with the lateral edge of the acromion, with a color code so that the tension band (white) in the middle is distinguished from the anchors (blue). The anterolateral portal is free. (L.H., long head.)

The spinal needle is again used to place the suture for the central TB with an intra-articular view, thanks to 2 successive passes in the space between the 2 anchor wires and medial to the rotator cable. A U-shaped passage is obtained (Figs 8 and 9A). The ALP is suture-free because the 6 strands are passed transcutaneously at the lateral edge of the acromion (Fig 9B).

**Fig 10:**
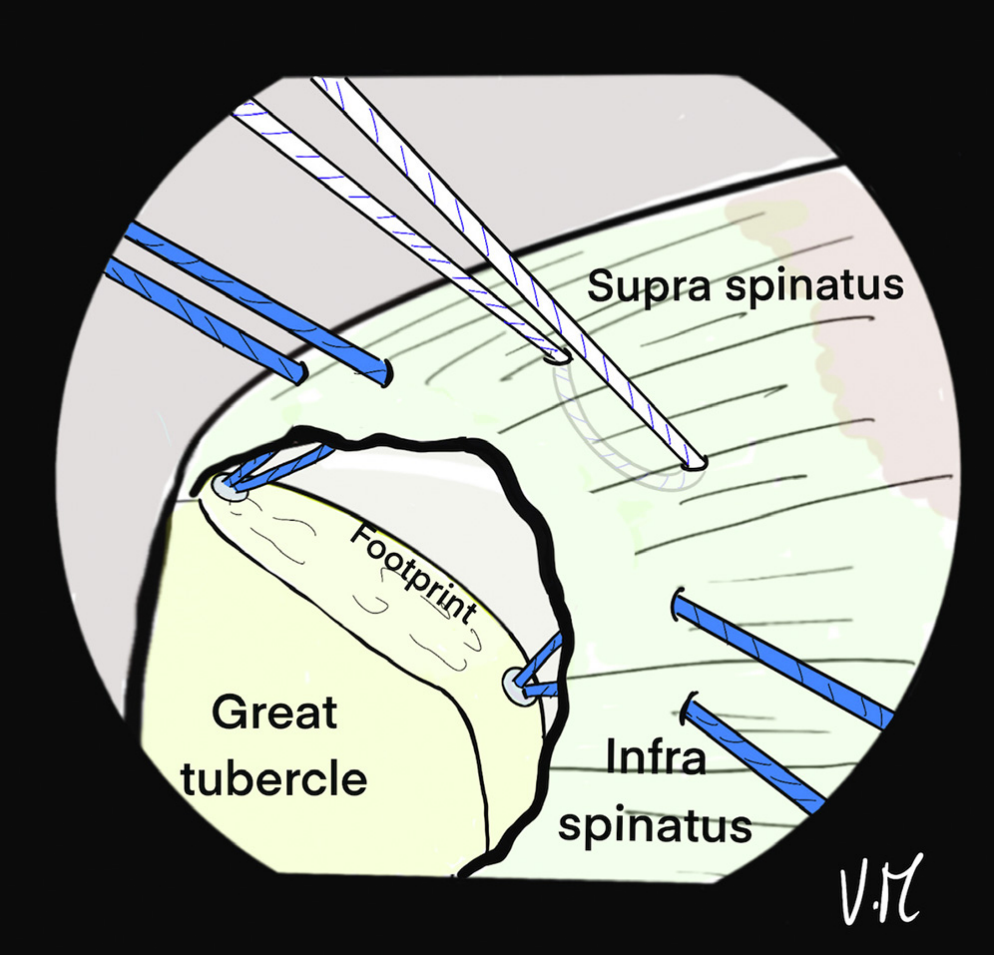
Drawing of an arthroscopic subacromial view from the posterior portal on a left shoulder in the beach-chair position. The surface of the bursal side of the cuff is visualized, as well as the 3 strands: Blue, anterior and posterior from the anchors. White, anchor-free for middle fixation. The passage of each of the strands is transcutaneous and transdeltoid.

**Fig 11:**
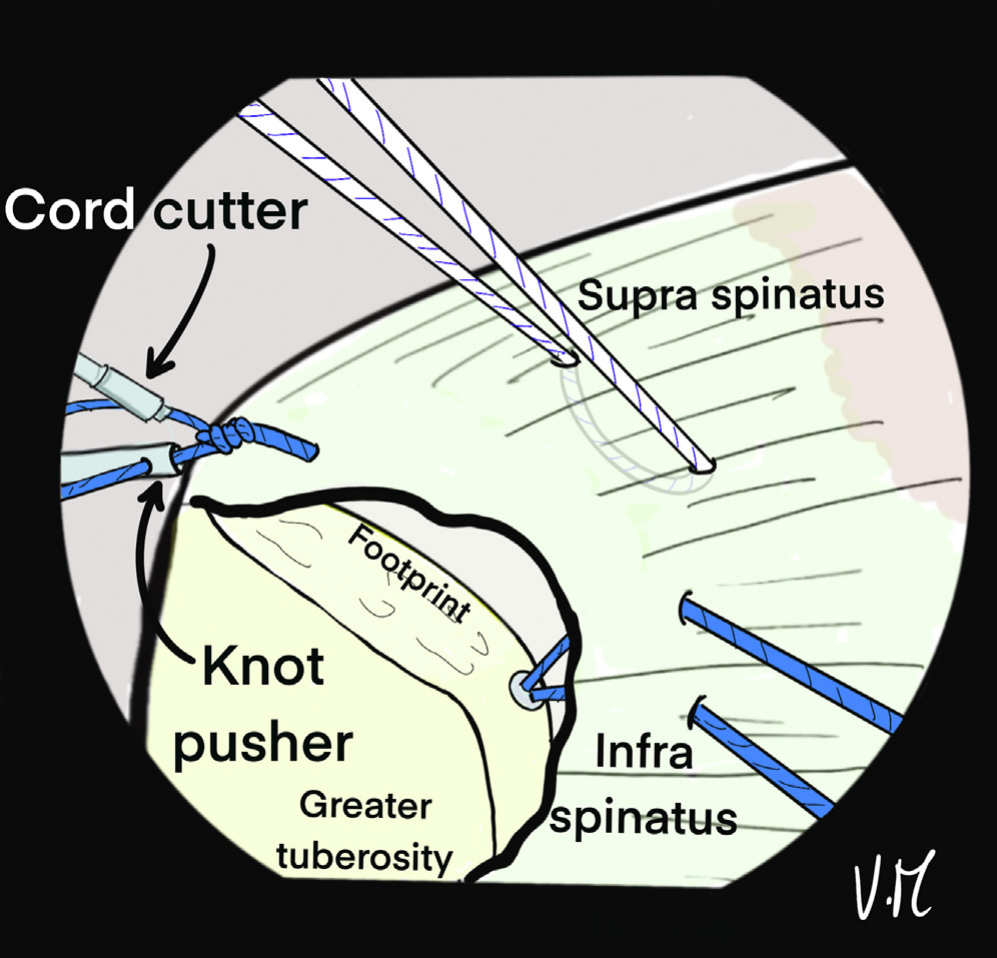
Drawing of an arthroscopic subacromial view from the posterior portal on a left shoulder in the beach-chair position. The 2 anterior blue strands are passed through the anterolateral portal with a suture retriever, then a surgeon's knot is made without excessive tension. To preserve the traction thread, the knot pusher is left on, and the cord cutter cuts the second strand (knotted) at the same time.

**Fig 12:**
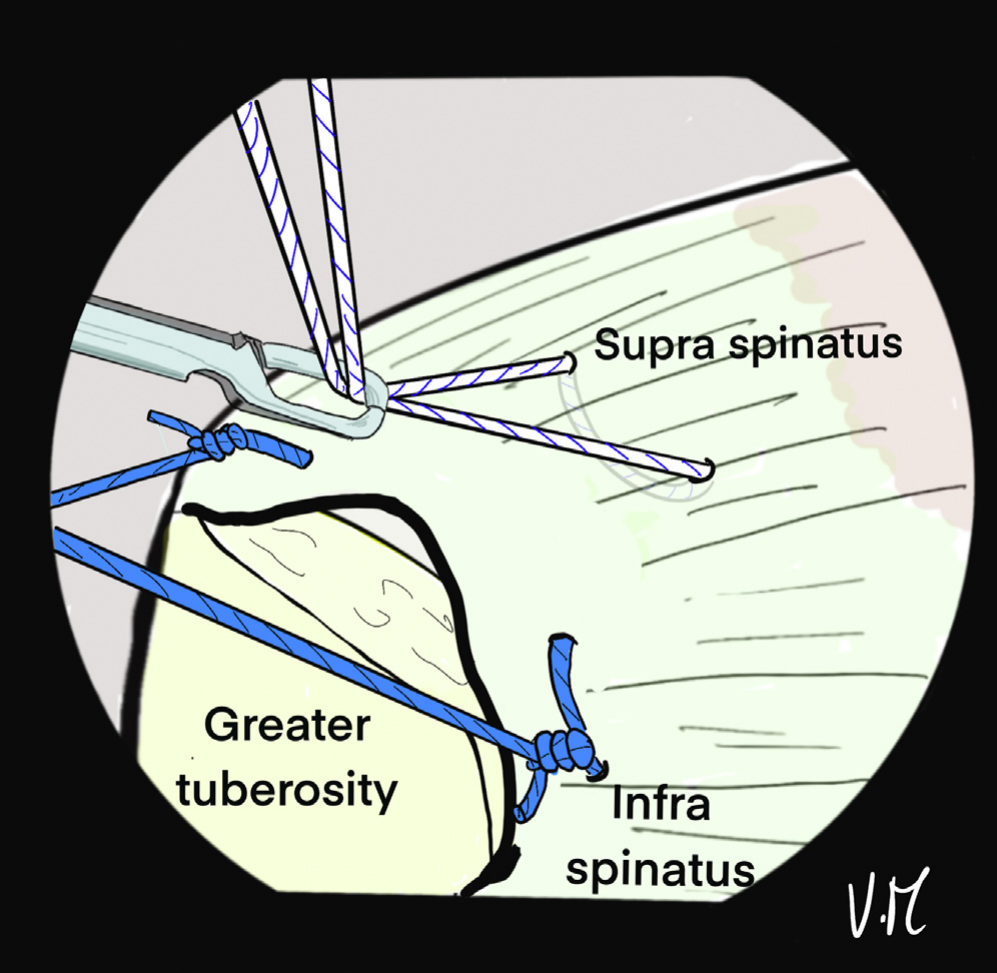
Drawing of an arthroscopic subacromial view from the posterior portal on a left shoulder in the beach-chair position. The same sequence is performed with the 2 posterior blue strands, keeping only the traction strand. A suture retriever is used to recover the 2 white strands through the anterolateral portal.

**Fig 13:**
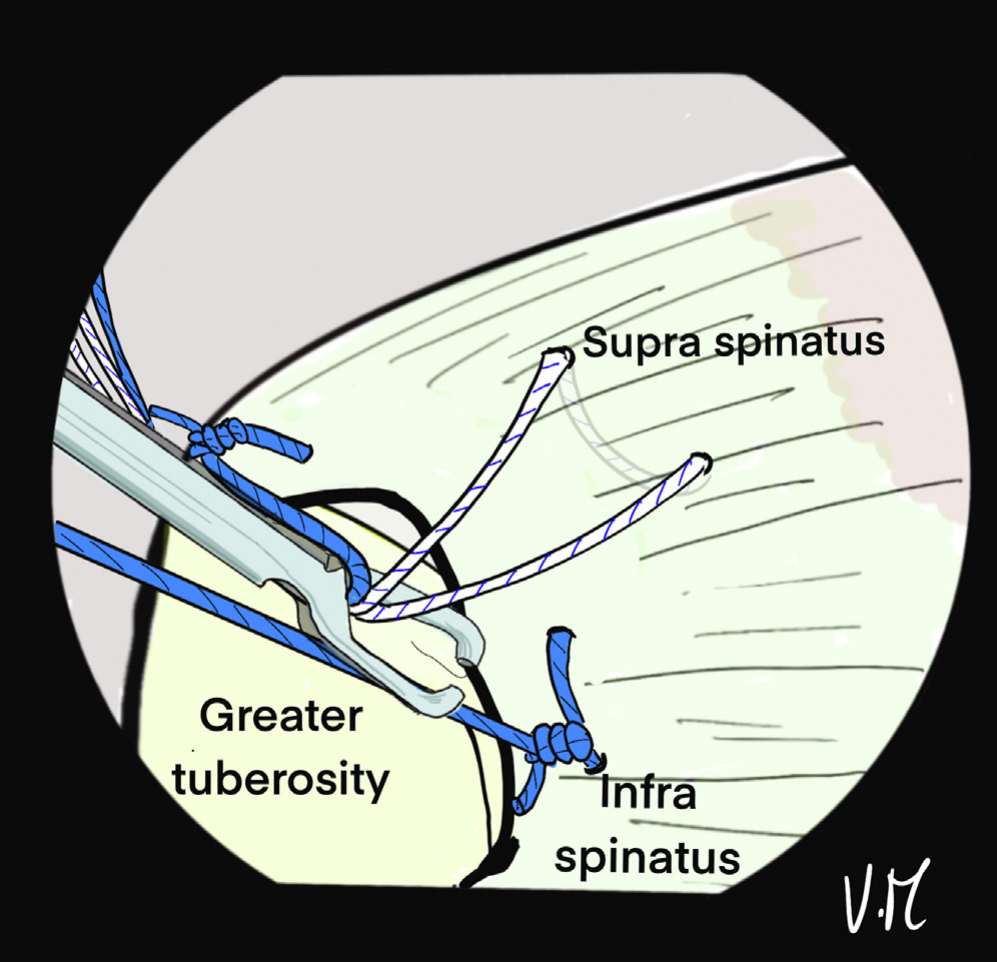
Drawing of an arthroscopic subacromial view from the posterior portal on a left shoulder in the beach-chair position. A suture grasper is passed through the anterolateral portal and retrieves the remaining 4 strands in a single pass.

The scope is then positioned in the subacromial space through the PP (Fig 10). The 2 strands of the anteromedial anchor are passed through ALP with a suture retriever and a surgeon’s knot is made on the most anterior strand. The knot pusher is left on the strand to cut the second strand (Fig 11). Then, the 2 strands of the posterior anchor are also retrieved through the ALP, and another knot is made on the posterior strand, which is retained, while the other strand is cut. Finally, the U-shaped suture is also passed through the ALP (Fig 12). The 4 remaining strands are recovered in a single pass with the suture retriever through the ALP: one main strand from each of the 2 anchors and the 2 strands of the U-shaped suture (Fig 13).

**Fig 14:**
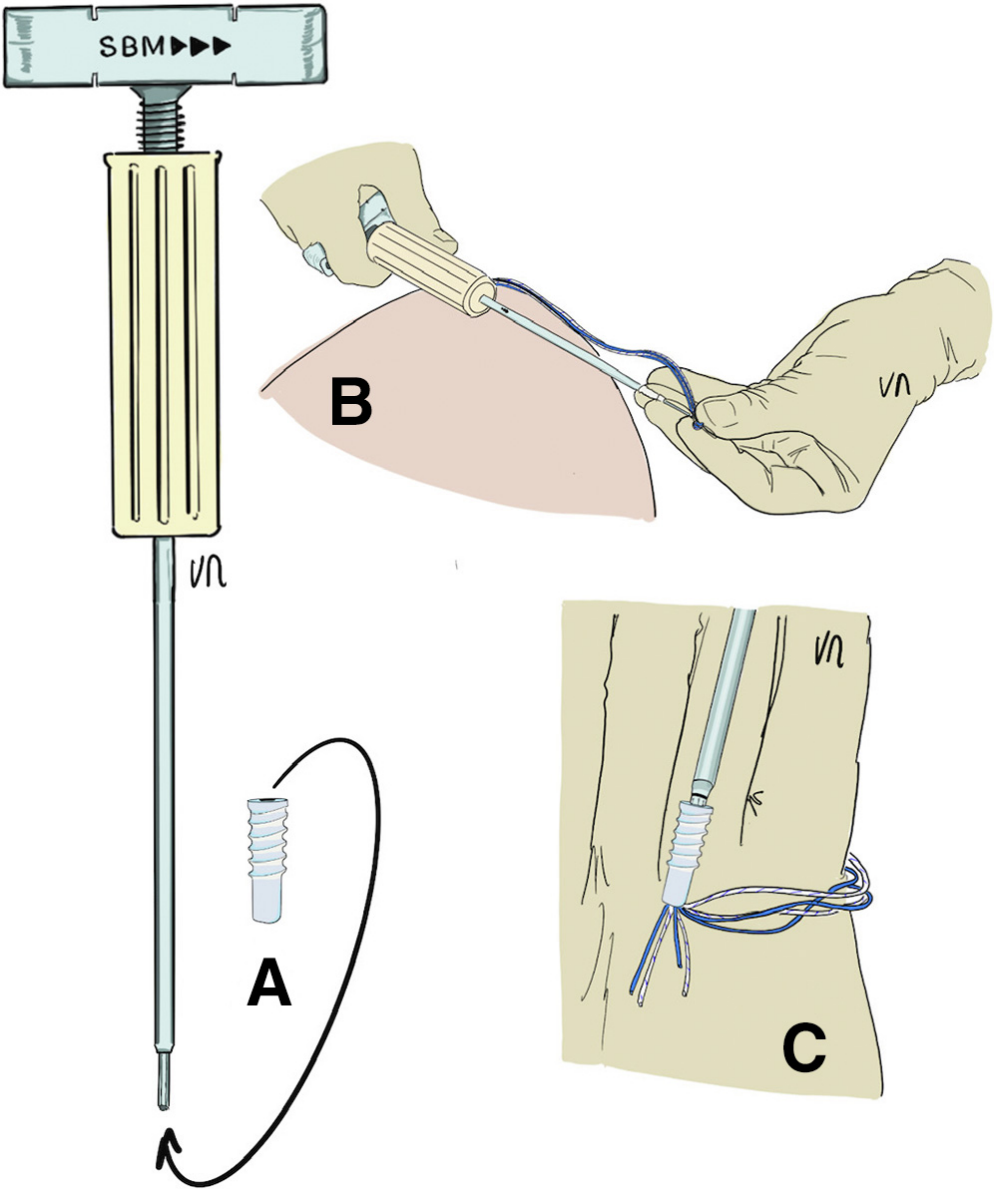
(A) A 5.5-mm FIXIT Knotless anchor (SBM; Lourdes, France) is removed from its package and fixed on a reusable screwdriver. (B) A reusable wire feeder is slipped into the screwdriver and the sutures are passed through its loop. (C) Four sutures may be passed inside the screwdriver with these reusable instruments.

**Fig 15:**
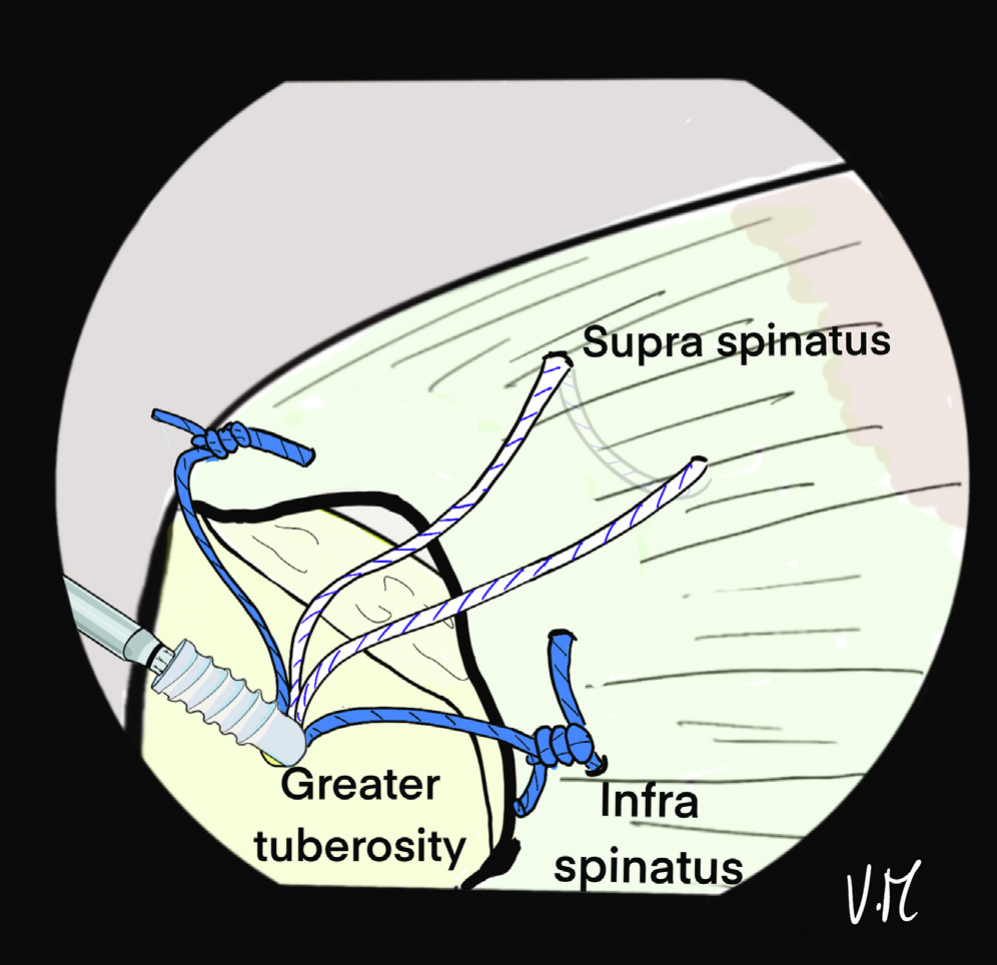
Drawing of an arthroscopic subacromial view from the posterior portal on a left shoulder in the beach-chair position. After placing and removing a 4.5-mm punch tap from the lateral aspect of the greater tuberosity, a 5.5-mm FIXIT Knotless (SBM; Lourdes, France) anchor is inserted over the first few millimeters. A smaller tap is used to optimize friction forces on the sutures.

The patient’s arm is repositioned with more abduction to expose and clean the lateral greater tuberosity. The 4.5-mm punch/tap is used perpendicular to the cortex. The 4 strands are passed through a 5.5-mm FIXIT Knotless anchor (SBM) with the reusable screwdriver (Fig 14). The knotless anchor is inserted and the 4 strands are tightened before fully tightening the screws (Fig 15). An anchor that is bigger than the tap should be used to optimize the forces of friction on the sutures. In case of poor bone quality when inserting the 4.5 punch/tap, the use of a 6.5-mm FIXIT Knotless anchor (SBM) is recommended in order to maintain a good interference with bone and sutures.

**Fig 16:**
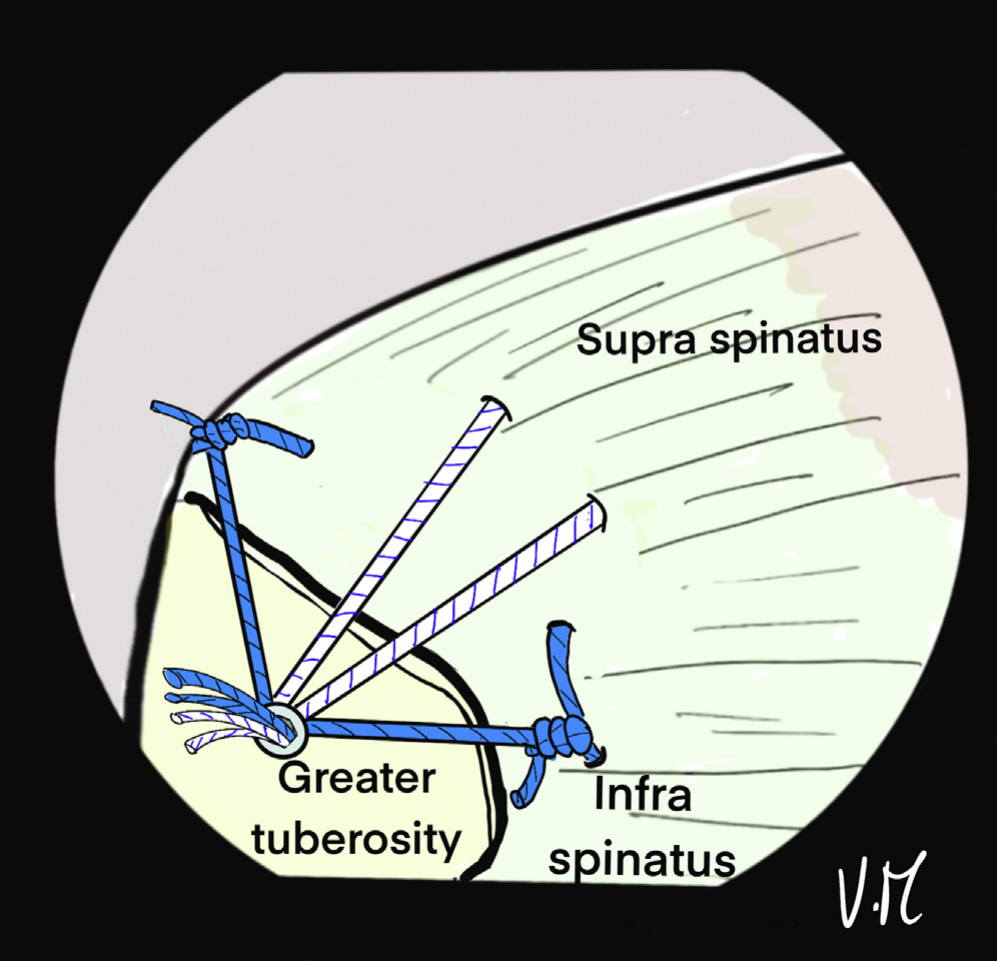
Drawing of an arthroscopic subacromial view from the posterior portal on a left shoulder in the beach-chair position. The 4 strands are pulled, one at a time without excessive tension and then the 5.5mm FIXIT knotless anchor (SBM; Lourdes, France) is screwed in until it is flush with the cortical bone. The screwdriver is removed and the sutures are is cut. The final view shows satisfactory reduction of the tendon with anterior and posterior fixation, and adequate homogenous bone/tendon contact thanks to the central tension band and the peripheral V-shaped suture bridge.

Correct assembly and reduction of the cuff are confirmed anterolaterally, posteriorly (Fig 16), and intra-articularly. If the cuff is too retracted or the critical shoulder angle is high, the use of a spinal needle is not possible. The Hybridge technique can still be carried out, using an automatic suture pass plier and working with subacromial view for the whole procedure.

### Postoperative Care

The patient is immobilized in a sling in internal rotation for 4 weeks during the day and 6 weeks at night, while performing self-rehabilitation exercises including active scapulothoracic mobilization and passive glenohumeral mobilization beginning the day after surgery, as well as rehabilitation with a physiotherapist 3 times a week.

## Discussion

Numerous repair techniques have been described for rotator cuff tears.^1^, ^2^, ^3^ The main clinical criteria for optimal healing include a low muscular fatty infiltration index (≤2)^7^^,^^8^ and moderate-to-intermediate tendon retraction.^8^ Other recent studies mention the harmful effects of tobacco,^9^ alcohol, and dyslipidemia.^10^ These should be the primary factors that are taken into consideration to obtain a high rate of healing (surgical repair techniques may be simplified if they are taken into account).

The Hybridge technique is mainly proposed to repair Colin type D lesions, which involve the entire supraspinatus tendon and part or all of the infraspinatus tendon.^6^ It also may be proposed for type C lesions by preserving the threads of the biceps tenodesis and repairing the superior tear of the subcapsular tendon. It is not indicated for isolated supraspinatus tendon lesions, as a TB with 1 or 2 anchors is usually sufficient in these cases, further limiting the cost and carbon impact. In contrast, in the case of severe posterior Colin’s type E lesions, more anchors are recommended.

Several scientific studies have reported the carbon impact and greenhouse gases generated by health care,^4^^,^^5^^,^^11^ in particular due to operating rooms.^12^^,^^13^ The main areas of improvement are the reduction of waste volumes and in the equipment supply chain.^14^, ^15^, ^16^ Surgeons play a primary role in reducing the surgical carbon footprint.^12^^,^^17^ Reducing the number of anchors used during surgery is simple and innovative as well as eco-responsible. The surgical technique we have described meets these criteria thanks to the following: (1) The use of only one second-row anchor, allowing easy passage of at least 4 strands rather than 2, like certain other anchor designs. (2) The use of a reusable screwdriver and wire feeder for this second row, limiting waste since no disposable screwdrivers are thrown away.^15^ Packaging is greatly reduced, limiting greenhouse gas emissions associated with the transportation and routing of the devices. (3) The use of a spinal needle and a monofilament suture to pass sutures through the tendon whenever possible, rather than automatic devices and other disposable systems. (4) Reduction of waste by reusing the suture of the posteromedial anchor to make the TB. (5) The use of 3 anchors instead of 4 or 5. This reduces both the direct costs and the carbon impact of surgery, as well as indirect costs by shortening the operating time, resulting in less electricity consumption in operating rooms and anesthesia, which has a very high greenhouse effect.^5^^,^^14^ Economically, this also contributes directly and indirectly to reducing the overall cost of procedures, which is a very sensitive issue in many countries.^13^^,^^18^

## Conclusions

The Hybridge repair technique is indicated for the treatment of extensive superior cuff tears. It combines the advantages of the suture-bridge and TB techniques. The use of a limited number of anchors and a reusable screwdriver, if possible, decreases the volume of greenhouse gases as part of a global approach to reducing the carbon footprint of health care worldwide.

## Supplementary Data

ICMJE author disclosure forms

Video 1 Intraoperative video demonstrating the Hybridge technique to repair larges posterosuperior tears of the rotator cuff. This is performed on a left shoulder, and the patient is in the lateral decubitus position, but scope is turned 90° to obtain a vertical view like in beach chair position. All sutures are first passed throw the tendons using the glenohumeral view. The cuff tear is then repaired with subacromial view, combining peripheral suture bridge and a central tension band with only 3 anchors.
